# Viral Encephalomyelitis

**DOI:** 10.1371/journal.ppat.1002004

**Published:** 2011-03-24

**Authors:** Diane E. Griffin

**Affiliations:** W. Harry Feinstone Department of Molecular Microbiology and Immunology, Johns Hopkins Bloomberg School of Public Health, Baltimore, Maryland, United States of America; University of California San Francisco, United States of America

## Encephalomyelitis Is an Unusual Complication of Infection with Encephalitic Viruses

Viral encephalomyelitis is an important cause of morbidity and mortality worldwide, and many encephalitic viruses are emerging and re-emerging due to changes in virulence, spread to new geographic regions, and adaptation to new hosts and vectors [1]. The term encephalomyelitis refers to inflammation in the brain and spinal cord that results from the immune response to virus infection. In humans, the viruses most commonly identified as causes of viral encephalomyelitis are herpesviruses and RNA viruses in the enterovirus (e.g., polio, enterovirus 71), rhabdovirus (e.g., rabies), alphavirus (e.g., eastern equine, Venezuelan equine, and western equine encephalitis), flavivirus (e.g., West Nile, Japanese encephalitis, Murray Valley, and tick-borne encephalitis), and bunyavirus (e.g., La Crosse) families. Other virus families with members that can cause acute encephalitis are the paramyxoviruses (e.g., Nipah, Hendra) and arenaviruses (e.g., lymphocytic choriomeningitis, Junin). However, this is certainly not a complete list, because for most cases of human viral encephalitis the etiologic agent is not identified, even when heroic attempts are made [2].

The primary target cells for most encephalitic viruses are neurons, although a few viruses attack cerebrovascular endothelial cells to cause ischemia and stroke or glial cells to cause demyelination, encephalopathy, or dementia [3]–[5]. Widespread infection of neurons may occur or viruses may display preferences for particular types of neurons in specific locations in the central nervous system (CNS). For instance, herpes simplex virus (HSV) type 1 often infects neurons in the hippocampus to cause behavioral changes, while poliovirus preferentially infects motor neurons in the brainstem and spinal cord to cause paralysis and Japanese encephalitis virus infects basal ganglia neurons to cause symptoms similar to those of Parkinson’s disease.

Because infections with encephalitic viruses are initiated outside the CNS (e.g., with an insect bite, skin, respiratory, or gastrointestinal infection), innate and adaptive immune responses are usually mounted rapidly enough to prevent virus entry into the CNS. Therefore, most viruses that can cause encephalitis more often cause asymptomatic infection or a febrile illness without neurologic disease, and encephalomyelitis is an uncommon complication of infection.

## Encephalitic Viruses Can Use Neuronal or Non-Neuronal Pathways to Enter the CNS

When a virus does invade the CNS, there are several routes by which infection of neurons can occur. The most common entry point is from the blood, and the level of viremia as a result of virus replication in peripheral organs often correlates with the likelihood of CNS infection. However, the blood–brain barrier (BBB), composed of vascular endothelial cells with tight junctions in contact with the foot processes of astrocytes, inhibits direct access to the brain parenchyma and neurons. Some neurotropic viruses can replicate in cerebrovascular endothelial cells, enter with infected leukocytes, or cross directly into the cerebrospinal fluid (CSF) through the porous capillaries of the choroid plexus. A specialized CNS entry pathway used by several viruses, most notably HSV, varicella zoster, and rabies viruses, is by way of nerve terminals in peripheral organs. These viruses can enter the nerve and then use neural transport mechanisms to transport the infecting virions to the neuronal cell body where replication occurs [6], [7]. A variation on this theme is infection through the exposed end processes of neurons in the nasal olfactory epithelium, followed by transport of the virus to the olfactory lobe within the CNS. Intranasal infection is commonly used to initiate infection of the CNS in experimental animals, but in humans this pathway may be important only in rare cases of aerosol exposure to a neurotropic virus [8]–[10].

In tissue culture systems most of these viruses can infect many types of cells, in addition to neurons. Few neuron-specific virus receptors have been identified (e.g., p75NTR, NCAM, and AChR for rabies; nectin for HSV) but these do not always account for neuronotropism in vivo [11]–[13]. Recent studies with HSV suggest that receptors used to enter processes of peripheral neurons can be different from those used to infect neurons in the brain [12]. Therefore, the mechanisms by which encephalitic viruses target neurons to the exclusion of other cells within the CNS are poorly understood. Once within the nervous system, encephalitic viruses often follow synaptic pathways for spread to other neuronal populations. These viruses interact with motor proteins either directly or through accessory proteins to travel using both anterograde (kinesin motors) and retrograde (dynein-dynactin motors) neuronal microtubule transport systems [6].

## Neuronal Damage Can Be Caused Directly by Virus Infection or by the Immune Response to the Infection

**Figure 1:**
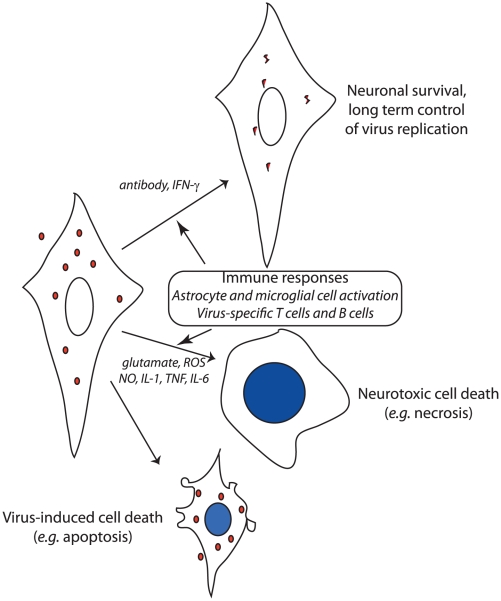
Schematic diagram of the potential outcomes after virus infection of neurons. Virus infection itself may induce death of the infected neuron, especially in young individuals. Neuronal death can also result from the local release of excitotoxic neurotransmitters (e.g., glutamate) and inflammatory mediators. Neurons can also survive infection and virus replication can be controlled through noncytolytic mechanisms such as local production of antiviral antibody and interferon (IFN)-γ. In the latter case, viral nucleic acid persists intracellularly and long-term immune control is necessary to prevent virus reactivation.

In addition to fever and headache, signs and symptoms of viral encephalomyelitis typically include evidence of neuronal dysfunction—seizures, cognitive impairment, ataxia, paralysis, etc. Virus replication can damage neurons directly by inducing cell death through apoptotic or necrotic mechanisms (Figure 1) [14]. Many viruses cause more severe CNS disease in the young. For these infections, immature neurons support more efficient virus replication and greater virus-induced cell death than mature neurons [15]. In humans, Venezuelan equine encephalitis and La Crosse viruses cause symptomatic neurologic disease almost exclusively in children, although adults are equally susceptible to infection [16], [17]. Conversely, for unexplained reasons, neurologic disease due to West Nile virus infection occurs primarily in people over the age of 60 years [18].

Although neuronal virus infection per se is necessary for, and contributes directly to, neuronal dysfunction, the inflammatory response in the CNS is also a major contributor to neuronal damage and can even result in death of nearby uninfected neurons (Figure 1) [19], [20]. The CNS inflammatory response to virus infection consists of activation and proliferation of resident astrocytes and microglial cells and of perivascular and parenchymal infiltration of activated monocytes and lymphocytes from the blood. The mechanism(s) by which the immune response causes neuronal damage are incompletely understood, but evidence exists for production of neurotoxins and reactive oxygen and nitrogen species by activated glial cells, increased levels of the excitotoxic neurotransmitter glutamate, and production of cytokines by activated lymphocytes [21]. The ability to control inflammation through induction of regulatory T cells and suppression of lymphocyte function by infected neurons can be an important determinant of outcome [22], [23]. In alphavirus encephalomyelitis, combined prevention of inflammation and glutamate excitotoxicity by treatment with glutamate receptor antagonists prevents paralysis and death despite continued virus replication [20].

## Virus Clearance from Neurons Is Complicated by the Irreplaceable Nature of Neurons

The immune response to infection can contribute to fatal disease, but is also necessary for recovery and virus clearance. Elimination of virus-infected cells from tissue requires elimination of all cells in which the virus is replicating. This can occur either by virus-induced or immune-mediated cytolysis. T cells are ideal for this purpose because they recognize viral antigen as processed antigen only in the context of cell surface–expressed MHC class I (CD8^+^ T cells) or of MHC class II (CD4^+^ T cells) and can possess cytotoxic properties. Neurons are long-lived essential cells that cannot be replaced, so a noncytolytic immune mechanism for virus clearance would be advantageous to the host to avoid death of surviving cells. If the immune clearance mechanism is damaging to the infected neuron, then the function of that neuron will be lost and the outcome for the host will be the same as if the virus infection had caused neuronal death.

Because mature neurons are relatively resistant to virus-induced cell death [15], noncytolytic mechanisms for virus clearance can be employed to control or eliminate infection. If infected cells are allowed to survive, the clearance of virus must include mechanisms for inhibiting intracellular synthesis of virus nucleic acid and protein, and for removing virus genomes from cells or preventing their replacement after degradation. Alphavirus encephalitis has been most thoroughly studied, and two noncytolytic clearance mechanisms have been identified: IFN-γ and anti-viral antibody (Figure 1). However, not all types of neurons are equally responsive to virus clearance by these mechanisms. IFN-γ acts through a Jak/STAT signaling pathway to activate an antiviral response that suppresses virus replication in motor neurons without toxicity, but the relevant antiviral proteins have not been identified [24]. Antibody to the E2 viral glycoprotein present on the surface of infected neurons suppresses virus replication in all populations of neurons through a pathway that requires bivalent antibody, but does not require complement or effector cells [25], [26].

## Recovery from Encephalitis Results in Virus Persistence and the Need for Long-Term Immune Control

Because the noncytolytic process for virus clearance does not completely eliminate viral RNA from neurons, a mechanism for long-term immunologic control of virus replication is needed to prevent virus reactivation or progressive disease [27], [28]. Antibody is likely to participate in control, as well as initial clearance. Maintaining adequate levels of antibody in the CNS for continued control of virus replication requires either passage of antibody from the blood into the brain parenchyma or local production by resident antibody-secreting cells. The BBB restricts the entry of proteins from the blood into the CNS, and although this function is compromised during the acute phase of infection, it is quickly repaired. Under normal conditions, levels of antibody in the brain are sustained at 1% of plasma levels that are likely to be inadequate for long-term prevention of virus reactivation. Therefore, resident long-lived antibody-secreting cells that can continue to produce antiviral antibody for a lifetime are a feature of recovery from most CNS virus infections [29]. Long-term immune control of virus replication is not always successful, leading to recurrent or progressive neurologic disease [30]–[32].

## Conclusions

Encephalomyelitis resulting from virus infection of neurons is a disease that can be fatal or result in permanent disability due to irreversible damage of infected neurons. The immune response to infection can enhance neuronal damage or can control virus replication by noncytolytic mechanisms and thus determine outcome. However, noncytolytic virus clearance results in persistence of viral nucleic acid in the CNS and thus establishes a need for long-term local immune responses to prevent reactivation of infection and progressive disease. Understanding these mechanisms is necessary for development of strategies for treating and preventing neurologic disease due to viral encephalomyelitis.
